# A remarkable synergistic effect at the transcriptomic level in peach fruits doubly infected by *prunus necrotic ringspot virus* and *peach latent mosaic viroid*

**DOI:** 10.1186/1743-422X-10-164

**Published:** 2013-05-28

**Authors:** Mari Carmen Herranz, Annette Niehl, Marlene Rosales, Nicola Fiore, Alan Zamorano, Antonio Granell, Vicente Pallas

**Affiliations:** 1 Instituto de Biología Molecular y Celular de Plantas, Universidad Politécnica de Valencia-Consejo Superior de Investigaciones Científicas, Avda. de los Naranjos s/n, Valencia 46022, Spain; 2 Botany, Department of Environmental Sciences, University of Basel, Hebelstrasse 1, Basel CH-4056, Switzerland; 3 Facultad de Agronomía e Ingeniería Forestal, Departamento de Ciencias Vegetales, Pontificia Universidad Católica de Chile, Av. Vicuña Mackenna 4860, Macul, Santiago 7820436, Chile; 4 Facultad de Ciencias Agronómicas, Departamento de Sanidad Vegetal, Universidad de Chile, Avenida Santa Rosa, Santiago 11315, Chile

**Keywords:** *Prunus persica*, Microarray, Mixed infections, Response to virus, Viroids, Synergistic effect

## Abstract

**Background:**

Microarray profiling is a powerful technique to investigate expression changes of large amounts of genes in response to specific environmental conditions. The majority of the studies investigating gene expression changes in virus-infected plants are limited to interactions between a virus and a model host plant, which usually is *Arabidopsis thaliana* or *Nicotiana benthamiana.* In the present work, we performed microarray profiling to explore changes in the expression profile of field-grown *Prunus persica* (peach) originating from Chile upon single and double infection with *Prunus necrotic ringspot virus* (PNRSV) and *Peach latent mosaic viroid* (PLMVd), worldwide natural pathogens of peach trees.

**Results:**

Upon single PLMVd or PNRSV infection, the number of statistically significant gene expression changes was relatively low. By contrast, doubly-infected fruits presented a high number of differentially regulated genes. Among these, down-regulated genes were prevalent. Functional categorization of the gene expression changes upon double PLMVd and PNRSV infection revealed protein modification and degradation as the functional category with the highest percentage of repressed genes whereas induced genes encoded mainly proteins related to phosphate, C-compound and carbohydrate metabolism and also protein modification. Overrepresentation analysis upon double infection with PLMVd and PNRSV revealed specific functional categories over- and underrepresented among the repressed genes indicating active counter-defense mechanisms of the pathogens during infection.

**Conclusions:**

Our results identify a novel synergistic effect of PLMVd and PNRSV on the transcriptome of peach fruits. We demonstrate that mixed infections, which occur frequently in field conditions, result in a more complex transcriptional response than that observed in single infections. Thus, our data demonstrate for the first time that the simultaneous infection of a viroid and a plant virus synergistically affect the host transcriptome in infected peach fruits. These field studies can help to fully understand plant-pathogen interactions and to develop appropriate crop protection strategies.

## Background

Plant viruses and viroids are obligate parasites that, consequently, depend on host factors to complete their life cycle. Subversion of host factors for viral replication and spread often disrupts cellular homeostasis and leads to pathogenesis and disease symptoms [1]. Among the most important consequences of viral pathogenesis are changes in the expression of host genes [2-4]. However, the functional significance of transcript changes in specific plant-pathogen interactions is still not well understood [2,5,6]. Thus, an improved understanding of these interactions should lead to new and creative methods to control plant virus/viroid diseases.

Stone fruit viruses and viroids can cause significant crop damage and crop losses [7]. Viroids are pathogens of food, industrial and ornamental plants. PLMVd, a member of the family *Avsunviroidae,* consists of a circular monocatenary RNA of 335-351 nucleotides and is distributed worldwide on peach [8]. Although PLMVd was initially considered as a latent pathogen it has been described that several isolates of PLMVd can cause deformation, discolored spots and cracked sutures in infected peach fruit [8]. PNRSV is a member of the genus *Ilarvirus* in the family *Bromoviridae*[9]*.* PNRSV has been reported to cause a reduction of 12% to 70% in tree growth and 5% to 70% yield losses depending on the cultivar [7,9].

Microarray technology is a powerful method to simultaneously evaluate changes in the expression of large amounts of genes in response to specific conditions. Currently, an increasing number of studies employ microarray profiling and de novo RNA sequencing to describe gene expression changes in virus/viroid-infected plants [10]. These studies reveal a significant impact of the viral/viroidal infection on host gene expression. Among the cellular processes affected upon infection are defense/stress responses, cell wall structure, chloroplast function, hormone signaling, protein metabolism, silencing and diverse other functions [2,5,10-17]. Moreover, infection leads to the modulated expression of various key regulators such as numerous transcription factors, antioxidants, metabolic enzymes and transporters [18]. However, the majority of studies analyzing gene expression changes upon infection are limited to specific host-pathogen interactions involving a given virus and a model host plant, usually *Arabidopsis thaliana* or *Nicotiana benthamiana*. Transcriptome analysis upon PNRSV infection, for example has only been performed in *N. benthamiana* plants [19]. PLMVd infection has not been investigated at the transcript level. Here, we explore and compare the gene expression profiles of peach, a natural host of PNRSV and PLMVd, upon single and double infection with the virus and the viroid in field conditions. Mixed infections of two pathogens occur in natural situations and have biological and epidemiological implications: frequently, simultaneous infections cause symptoms with a higher severity than those induced in single infections by either of the two pathogens. This phenomenon is known as synergism in pathology [20] and has been mainly studied between two different viruses as, for example, mixed infection of *Potato virus X* (PVX) and *Potato virus Y* (PVY) [21-24].

Double infection of peach with PNRSV and PLMVd frequently occurs in the field. Although the absence of symptoms was a common characteristic for all the samples analyzed in this study, we wondered whether the double infection with PNRSV and PLMVd could affect host gene expression differently than single infection. To approach this question, we conducted microarray profiling of peach fruits originating from Chile, singly and doubly infected with PNRSV and PLMVd using microarray slides containing 4,261 unigenes obtained from mesocarp and exocarp tissues of two full-sib progeny contrasting for chilling injury (CI) [25]. Our results clearly demonstrate, for the first time, a synergistic effect at the transcriptomic level between a viroid and a plant virus. The synergistic effect is represented by considerably increased amplitude of differentially expressed genes. The functional identity of those genes with significant alterations upon viral and/or viroidal infection and their putative role in the disease process are discussed.

## Results

### Sampling and experimental design

Between 2006 and 2008 a number of orchards of different size were visited to assess their sanitary status (Fiore et al., unpublished data) in austral hemisphere spring (September to November) in Chile. Fruits of a total of 2,456 peach trees were sampled during the whole survey period and tested for the presence of viruses and viroids known to affect stone fruit plants in Chile. Among others, samples were tested positive for PNRSV and *Prune dwarf virus* (PDV), which are the viruses most frequently encountered in southeastern peach, *Tomato ringspot virus* (TomRSV), *Plum pox virus* (PPV) and PLMVd. Interestingly, the analysis also revealed that the most recurrent mixed infection was PLMVd-PNRSV (Fiore et al., unpublished data).

**Figure 1:**
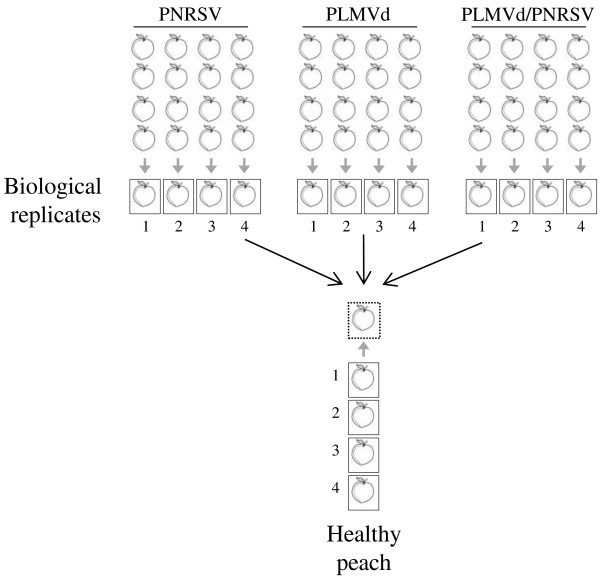
**Experimental design for microarray hybridizations.** Samples tested positive for either PNRSV, PLMVd, both pathogens simultaneously, or healthy controls were analysed in four independent biological replicates and two technical replicates (dye swaps: Cy3-healthy/Cy5-infected or Cy3-infected/Cy5-healthy). Each biological replicate (numbered 1 to 4) for each infection and healthy plants consisted of a pool of four infected peach fruits from four different trees. The different sets of probes were hybridized to a cDNA microarray representing all the unigenes in the ChillPeach database. In detail, microarray hybridizations were performed by hybridizing each virus infected biological replicate and the healthy control replicate to an array resulting in a total of 16 hybridizations. Dye swaps were performed for two of the four replicates of each infection.

For microarray analysis, peach fruits infected either with PNRSV, PLMVd or simultaneously with both pathogens were collected at developmental stage (S4), at approximately 100 days after boom, period where the fruit reaches the final full size and enters the fruit ripening or climacteric stage according with the description provided by Zanchin et al. [26]. The tissue used for RNA extraction was exocarp and mesocarp. The analysis of the PLMVd sequences recovered from the infected trees revealed a mix of quasispecies (e.g. PLMVd-4tun, ZZ46, lib P7, PC-C29 etc.) none of them associated to symptomatic strains. For the PNRSV all the sequence variants recovered belong to the PV32 group for which no specific symptomatology has been observed [9]. To obtain robust and statistically accurate data, four biological replicates and two technical, dye swapped replicates of healthy, virus infected, viroid infected, and doubly infected samples were analyzed. Each biological replicate consisted of a pool of four peach fruits from four different trees previously tested positive (for the infected samples) or negative (for the healthy controls) (Figure 1). RNA of the different sets of samples was hybridized to cDNA microarrays representing all the unigenes in the ChillPeach (http://bioinfo.ibmcp.upv.es/genomics/ChillPeachDB) database.

### Comparison of the PNRSV and PLMVd concentrations in infected peach fruits

**Figure 2:**
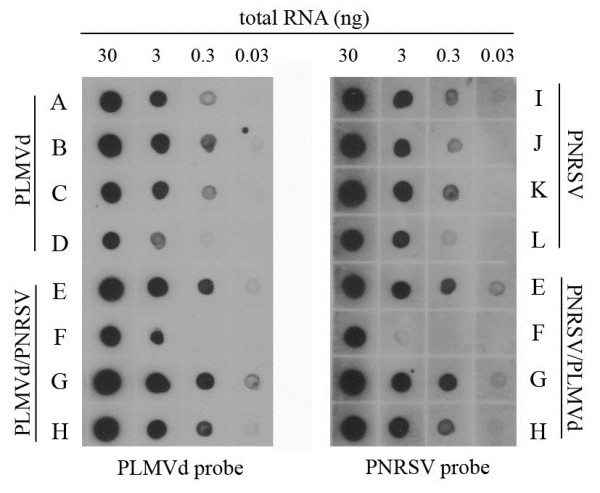
**Comparison of the amount of virus/viroid in infected plants by non-isotopic molecular hybridization.** Crude extracts of infected peaches (capital letters from A to H) were dotted onto nylon membranes in 10-fold dilutions and hybridized to PNRSV and PLMVd probes. Numbers at the top of the figure represent the amount of analyzed RNA in nanograms (ng). Chemiluminiscent detection was carried out after 15- min exposure.

To determine the concentration of PNRSV and/or PLMVd in peach fruits, serial dilutions of each total RNA preparation were blotted onto a nylon membrane. The RNA samples were analyzed using specific digoxigenin-labelled riboprobes [27,28]. The concentration of either PLMVd or PNRSV (Figure 2: samples A, B, C, D and I, J, K and L respectively) was very similar among the singly infected samples reaching in both cases a detection limit of 0.3 ng/μL. In the mixed infections (samples E, F, G and H) a slight increase in the accumulation of both pathogens was observed (Figure 2 and Additional file 1: Figure S1).

### Cumulative changes in gene expression

**Figure 3:**
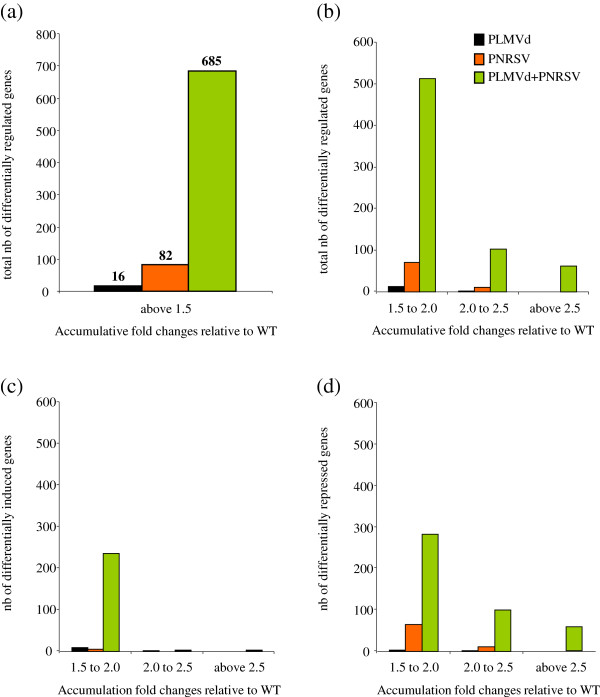
**Number of genes with significant expression changes upon infection.** The number of genes with significant expression changes upon infection is shown for PLMVd (black bars), PNRSV (orange bars) or PLMVd-PNRSV (green bars) infected peach fruits. (**a**) Total number of differentially regulated genes with accumulative fold changes above 1.5. (**b**) Total number of differentially regulated genes with accumulative fold changes in three different intervals: 1.5 to 2.0, 2.0 to 2.5 and above 2.5. (**c** and **d**) Total number of differentially induced and repressed genes respectively, with accumulative fold changes between 1.5 and 2.0, 2.0 and 2.5 and above 2.5.

**Figure 4:**
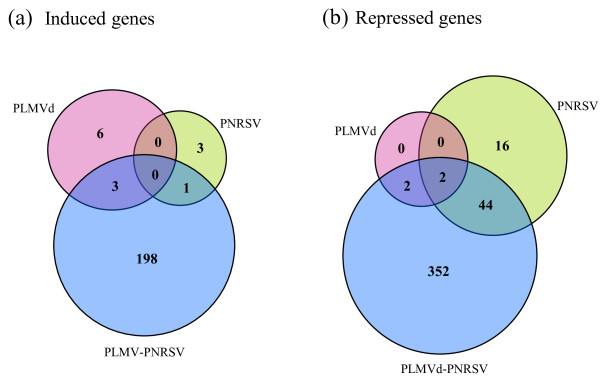
**Venn diagrams displaying the overlap in differentially regulated genes upon single or double infection with PLMVd or PNRSV.** Overlap in differentially induced (**a**) and repressed (**b**) gene sets upon infection with PNRSV, PLMVd or both. Numbers shown in the nonintersecting segments represent the number of genes with statistically significant expression changes unique to each infected sample whereas numbers within intersections represent the number of genes with statistically significant expression changes occurring in common upon infection with PLMVd, PNRSV, or double infection.

Microarray analysis of the samples was performed using the ChillPeach array containing 4,261 peach unigenes. The number of genes with statistically significant expression changes of at least 1.5 fold relative to healthy plants was 16 and 82 for fruits infected with PLMVd and PNRSV, respectively (Figure 3a). Out of these genes, 13 and 66 have orthologs in *A. thaliana*. Interestingly, fruits simultaneously infected with both pathogens presented a significantly higher number of differentially regulated genes (685, out of which 602 have orthologs in *A*. *thaliana*). The majority of differentially regulated genes exhibited fold changes between 1.5 and 2.0 (Figure 3b) upon infection, however, in contrast to singly, doubly infected fruits displayed a considerable number of genes with fold changes above 2. Comparison of induced and repressed genes revealed similar results as the analysis of the total number of genes (Figure 3c and d). Moreover, this analysis revealed that virus, viroid, and double infection with both pathogens mainly lead to the repression of host gene expression. In detail, approximately 70% of genes differentially regulated upon PNRSV, PLMVd, or double infection, respectively were downregulated, while the remaining 30% were induced (Figure 3c and d). Out of the genes with orthologs in Arabidopsis only a few were induced in PNRSV (4 genes) and PLMVd (9 genes) infected fruits whereas 202 genes exhibited increased expression levels compared to the healthy control in peaches infected with both pathogens (Figure 4a and Additional file 2: Table S1). 400, 62 and 4 genes with orthologs in Arabidopis displayed significantly reduced expression upon infection, in doubly, PNRSV and PLMVd infected samples, respectively (Figure 4b and Additional file 2: Table S1). Interestingly 70% (44 genes) of the PNRSV infected genes were found in PNRSV-PLMVd double infection (meaning 10% for double infected peaches), thus indicating that PNRSV and PNRSV-PLMVd infection deregulate a largely similar set of genes.

Taken together, transcript analysis revealed a higher number of repressed than induced genes upon infection. In addition, double infection with PNRSV and PLMVd resulted in many more expression changes compared to single infection with either pathogen. Thus, PNRSV and PLMVd appear to exhibit a synergistic effect with respect to the regulation of host gene expression.

### Validation of expression changes by quantitative RT-PCR

**Figure 5:**
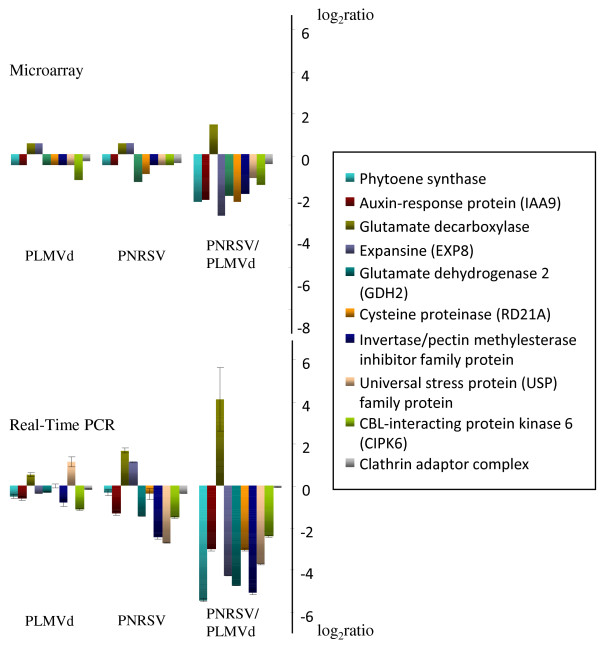
**Validation of microarray data using quantitative RT-PCR.** The Microarray (upper graph) and quantitative RT-PCR (lower graph) data are shown for nine randomly selected genes with statistically significant expression changes in the microarray experiments: *Glutamate dehydrogenase 2 (GDH2)* (ppa006458m.g)*, Cysteine proteinase (RD21A)* (ppa005328m.g)*, Invertase/pectin methylesterase inhibitor family protein* (ppa011831m.g)*, Universal stress protein (USP) family protein* (ppa012560m.g)*, CBL-interacting protein kinase 6 (CIPK6)* (ppa005365m.g), *Phytoene synthase* (ppa005962m.g), *Auxine response protein (IAA9)* (ppa007194m.g), *Glutamate descarboxilase* (ppa004796m.g) and *Expansin (EXP8)* (ppa010260m.g). *Clathrin adapter complex* (ppa005912m.g) was used as a control gene with unchanged expression upon infection. Values represent the log_2_ratio.

As reference genes for quantitative RT-PCR we chose peach orthologs of Arabidopsis F-box family protein (F-BOX) and elongation factor 1-α (EF1-α), which were previously reported to exhibit stable expression upon virus infection in Arabidopsis [29] and the clathrin adaptor complex (CAC) as internal control of the microarray. All reference genes exhibited similar expression levels in virus, viroid infected or healthy peach fruits, demonstrating their suitability as reference genes in peach. To validate the results obtained by microarray analysis we performed quantitative RT-PCR with nine randomly selected genes displaying induced or reduced expression upon infection plus the CAC. The nine selected genes were *Phytoene synthase, Auxin response protein (IAA9), Expansin (EXP8), Glutamate descarboxilase, Glutamate dehydrogenase 2 (GDH2), Cysteine proteinase (RD21A), Invertase/pectin methylesterase inhibitor family protein, Universal stress protein (USP) family protein, CBL-interacting protein kinase 6 (CIPK6) and Phosphate-responsive protein, putative (E).* Out of these nine genes analyzed, seven displayed consistent gene expression changes with both methods (Figure 5). *Phytoene synthase*, *Auxin response protein, Glutamate descarboxilase, Glutamate dehydrogenase 2 (GDH2), Cysteine proteinase (RD21A), Invertase/pectin methylesterase inhibitor family protein, CBL-interacting protein kinase 6 (CIPK6)* exhibited reduced expression in PNRSV, PLMVd and PNRSV-PLMVd infected samples compared to the healthy control plants, and *Glutamate decarboxylase* was induced upon infection. As already seen by microarray analysis, all expression changes were more pronounced upon double infection with PNRSV and PLMVd. Expression levels for *Expansin*, however, differed in Microarray and quantitative RT-PCR experiments. While the gene was upregulated in microarray analyses, it was downregulated by qRT-PCR in PNRSV infected samples. Expansins exist in a multigene family in Arabidopsis [30]. It is likely, that also peach has several expansin isoforms. Thus, the differences in expression of Expansin upon infection may result from several isoforms recognized by the cDNA array and the specific detection of only one isoform in our quantitative RT-PCR experiments. In a similar way, expression levels for *Universal stress protein (USP) family protein* are distinct in the two different analysis. A similar situation to that observed for Expansins occurs with this gene and thus, the same explanation could be valid for this protein family. Taken together, the results of qRT-PCR analyses confirmed the gene expression changes seen by microarrays analysis.

### Functional categorization of genes with induced and repressed expression upon double infection with PNRSV and PLMVd

**Figure 6:**
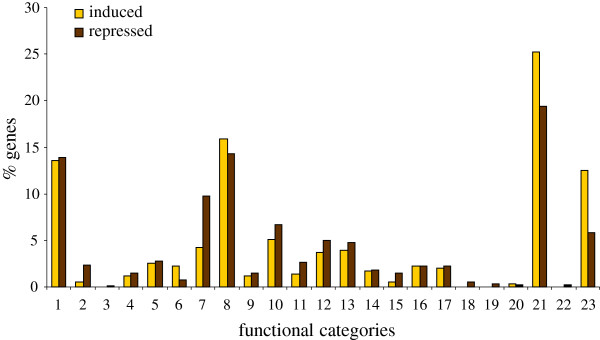
**Functional category distribution of the differentially expressed genes upon PNRSV-PLMVd infection.** The graph shows the distribution (in %) of genes with induced (in yellow) and reduced (in brown) expression upon double infection into different functional categories present in the MIPS Functional Catalogue Database (FunCatDB).

As stated above, a clear synergistic effect on the peach transcriptome was observed in samples simultaneously infected with both pathogens. To gain insight into functions of genes exhibiting modulated expression upon infection we grouped the genes with significantly modulated expression upon double infection with PNRSV and PLMVd into 23 functional categories using the Munich Information Center for Protein Sequences Functional Catalogue Database (MIPS FunCatDB, http://mips.helmholtz-muenchen.de/proj/funcatDB/) (Figure 6). Most repressed or induced genes can be assigned to three functional categories: “metabolism”, “protein with binding function or cofactor requirement” and “subcellular localization”. “Protein modification and degradation” was the functional category to which most repressed genes could be assigned, whereas induced genes were mainly predicted to encode proteins in the category “related to phosphate, C-compound and carbohydrate metabolism” and also “protein modification” (Table 1).

**Table 1:** Gene distribution of the three functional categories with the highest number of statistically significant genes according to MIPS Functional Catalogue Database

	**Induced**	**Repressed**
	%	%
*Subcellular localization*		
cell wall	3	1
plasma membrane/membrane attached	5	2
cytoplasm	9	4
cytoskeleton	1	0
endoplasmic reticulum	3	1
golgi	1	0
intracellular transport vesicles	2	1
nucleus	13	6
mithocondrion	31	14
plastid	32	15
*Protein fate*		
protein folding and stabilization	20	10
protein targeting sortening and translocation	7	0
protein modification	60	51
assembly of protein complexes	7	0
protein/peptid degradation	7	39
*Metabolism*		
aminoacid metabolism	5	10
nitrogen, selenium and sulfur metabolism	5	4
nucleotide/nucleoside/nucleobase metabolism	6	9
phosfate metabolis	19	29
c-compound and carbohydrate metabolism	35	26
lipid, fatty acid and isoprenoid metabolism	14	11
metabolism of vitamins, cofactors and prosthetic groups	5	3
secondary metabolism	11	9

### Statistical analysis of functional categories

To identify overrepresented functional categories among induced or repressed genes, we subjected genes to analysis by Babelomics 4.2 FatiGO and single enrichment analysis (SEA). FatiGO uses Fisher’s exact test for 2×2 contingency tables to check for significant over-representation of GO terms. Singular enrichment analysis identifies enriched GO terms in a list of microarray probe sets or gene identifiers. We used this test to identify over- or underrepresented GO terms in the set of genes with significant changes upon infection with both pathogens compared to the whole set of genes represented on the microarray. This analysis should enable us to predict a role of a certain biological processes in response to infection rather than merely calculate a number of up-regulated and down-regulated genes.

For over-representation analysis, each gene was assigned to one of the three main gene ontologies (GO): Biological Process, Cellular Component and Molecular Function. For the 198 up-regulated genes unique to double infection, no functional category was over- or under- represented with respect to the functional category distribution in the microarray. Table 2 shows the non-redundant functional categories significantly over- and under-represented among those genes that were significantly down-regulated upon double infection. The enrichment analysis resulted in a total of 6, 7 and 5 functional categories in GO Biological Process, Molecular Function and Cellular Component respectively. To estimate the number of times that a certain functional category was over- or under- represented, we calculated the ratio between the percentage of genes in each functional category and the percentage of genes corresponding to the same functional category in the whole array (Table 2). We found higher ratios for “alcohol metabolic process” and “response to cadmium ion” in GO Biological Process, “oxidoreductase activity, acting on NADH or NADPH” in GO Molecular Function and “peroxisome” in GO Cellular Component.

**Table 2:** Non-reduntant GO categories identified as enriched in down-regulated genes in double infected plants

**352 diferential genes, >1.5 fold**						
		**list1**	**list1 percentage**	**ratio**	**list2**	**list2 percentage**
***Biological Process***						
GO:0006066	Alcohol metabolic process	21	6.0	2.5	93	2.4
GO:0006508	Proteolysis	35	9.9	1.8	2.1	5.3
GO:0006950	Response to Stress	59	16.8	1.6	386	10.1
GO:0030163	Protein catabolic process	36	10.2	1.9	203	5.3
GO:0046686	Response to cadmiun ion	35	9.9	3.2	119	3.1
GO:0055114	Oxidation reduction	38	10.8	2.0	191	5.0
***Molecular Function***						
GO:0001882	Nucleoside binding	65	18.5	1.6	434	113
GO:0005524	ATP binding	60	17.1	1.6	410	10.7
GO:0043169	Cation binding	76	21.6	1.5	532	13.9
GO:0046872	Metal ion binding	73	20.7	1.5	511	13.4
GO:0005515	Protein binding	81	23.0	1.4	623	16.3
GO:0050662	Coenzyme binding	18	5.1	2.4	79	2.1
GO:0016651	oxidoreductase activity, acting on NADH or NADPH	10	2.8	4.0	26	0.7
***Celular component***						
GO:0005886	Plasma membrane	73	20.7	1.6	503	13.1
GO:0005829	Cytosol	33	9.4	1.7	206	5.4
GO:0005773	Vacuole	28	8.0	1.9	159	4.2
GO:0005777	Peroxisome	10	2.8	3.5	30	0.8
GO:0048046	Apoplast	16	4.6	2.8	61	1.6
**150 genes diferenciales, >2 fold**						
		**list1**	**list1 percentage**	**ratio**	**list2**	**list2 percentage**
***Biological Process***						
GO:0006520	Cellular amino acid metabolic process	12	8.0	3.0	102	2.7
GO:0009308	Amine metabolic process	14	9.3	3.0	118	3.1
GO:0009414	Response to water deprivation	9	6.0	5.5	41	1.1
GO:0009628	Response to abiotic stimulus	24	16.0	2.3	265	6.9
GO:0046686	Response to cadmium ion	14	9.3	3.0	119	3.1
GO:0055114	Oxidation reduction	18	12.0	2.4	191	5.0
***Molecular Function***						
NO GOs						
***Celular component***						
GO:000577	vacuole	19	12.7	3	159	4.2

The absolute number of genes belonging to each category is specified in list1 (differentially expressed genes) and list2 (total number of genes in the whole microarray). Ratio represents the number of times that one category is enriched. The analysis was performed for the genes with a fold change value above 1.5 (upper table, 352 genes) and above 2.0 (lower table, 150 genes).

To gain a more precise idea about functional classes of proteins with altered expression upon double infection with PNRSV and PLMVd, we increased the threshold of genes subjected to over-representation analysis to at least 2-fold changes in expression level. With these settings, the majority of the functional groups over- represented within GO Biological Process was “related with cellular amino acid metabolic process” and “response to abiotic stresses”. Interestingly, “response to water deprivation” was overrepresented five times compared to the normal distribution of this functional class on the array (Table 2). No functional category was over- or under- represented in GO Molecular Function and only one category with 19 genes, “vacuole”, was overrepresented in GO Cellular Component.

## Discussion

Viroids and viruses depend on host components to complete their life cycle and thus interfere with host processes, such as host gene expression during infection. The identification of genes with altered expression in response to a certain virus/viroid infection can provide clues about the viral life cycle and requirements which need to be met by the host cell. Moreover, a better understanding of host responses to infection facilitates the development of methods to control pathogen infection. To date, the effects of viroid infection on the host transcriptome have been reported for four viroids. These studies used PCR-based cDNA library subtraction [15]; differential display RT-PCR and quantitative real-time RT-PCR [31,32] or cDNA microarray [17,33] to detect host gene expression changes. Currently, a number of studies explore gene expression changes in virus-infected plants using DNA microarrays. All these studies involve a given virus and a model host plant, usually *A. thaliana*[25] or *N. benthamiana*[19]. In the present work, we collected peach fruits, a natural host of both PNRSV and PLMVd, at development stage (S4) and studied changes in their transcriptome upon infection with the two pathogens. Today, no array including the whole peach fruit genome is available. Thus, we used a cDNA microarray containing probes for 4,261 genes expressed during chilling injury (CI) development [34].

During infection, the concentration of PNRSV and PLMVd in fruits is significantly higher (100:1) than in leaves [35,36]. In addition, the occurrence of peach trees doubly infected with both, PNRSV and PLMVd in the field is considerable (e.g. Fiore et al., unpublished data); [37]. Here, we compared transcript changes in asymptomatic singly and doubly PNRSV or PLMVd infected peach fruits. Overall, our gene expression analysis revealed a relatively low number of differentially expressed genes in single PNRSV infections compared to the healthy control plants (82 genes). This is consistent with the results obtained by Dardick [19] in which only 89 genes were significantly differentially expressed in PNRSV-infected *N. benthamiana* plants compared to mock controls. Peach fruits infected with PLMVd also mounted only a moderate response at transcript level (16 genes). The PLMVd variants detected in our study are all considered as latent. In future studies it would be interesting to compare the host transcripts changes caused by variants causing the “calico” [8] syndrome with those caused by milder variants. PNRSV infected samples were also asymptomatic and all the sequence variants recovered belonged to the PV32 group [9]. Remarkably, in the doubly infected peach fruits a significant synergistic effect on the host transcriptome was observed. Considering the three different infections, the total number of genes with significantly altered expression (at least 1.5 fold change in expression level) was 783, which represents 18% of the whole array. From these 783 genes and eliminating those with significant expression changes occurring in common upon the three scenarios, 627 have orthologs in Arabidopsis (Additional file 3: Table S2). Among those genes 211 exhibited induced and 416 reduced expression. Thus, the total number of genes with reduced expression exceeded that of genes with induced expression by two-fold. This is surprising as comparative analysis of the Arabidopsis transcriptome during compatible interaction with plant viruses [25] revealed that there was a greater number of up-regulated than repressed genes in the course of viral pathogenesis. However, the authors demonstrated that each virus-host interaction is unique in terms of the genes with altered expression levels and to find a common pattern among different viruses is difficult [25]. In addition, in one of the few studies in which a temporal analysis after virus/viroid infection was carried out, Rizza et al., [17] showed that the pattern of up-regulated vs down regulated genes can change in pre-symptomatic when compared to post-symptomatic stages of Etrog citron infected with Citrus exocortis viroid (CEVd).

The overlap in significantly altered gene expression among the two single and the double infections was low. Common genes that were differentially expressed during each infection scenario are shown in Additional file 4: Table S3. This is not surprising considering the small number of genes with significant changes in both single infections. Consistently, Postnikova and Nemchinov [25] have recently shown that the number of host genes commonly affected during infection of Arabidopsis with either of twelve different viruses studied is very limited. Due to the nature of this specialized database it is difficult to establish a relationship between genes involved in peach ripening and those affected by the pathogens. Nevertheless in our study we observed some genes previously described as related with ripening with altered expression. Most of them did it in the, *a priori*, unexpected wayside slowing down the ripening (see Additional file 5: Table S4 and references [38-41]). Although we did not follow with great detail whether or not mixed infections had modified the ripening it is noteworthy to emphasize that alterations in ripening date have been described in several virus-host interactions regarding fruit trees (see [42] for review).

The higher number of genes significantly altered exclusively upon double infection with PLMVd and PNRSV led us to investigate whether some specific functional categories were over- or under- represented among those genes. Interestingly, among the 198 significantly induced genes unique to double infection (Figure 4) none of the functional categories appeared to be enriched in comparison with the normal distribution of genes in the whole microarray. As the array used in this study is enriched with sequences of genes that are implicated in chilling injury development, the lack of overrepresentation of functional categories among the induced genes upon infection compared to the genes represented on the array may indicate a similarity between the functional classes induced upon the two types of stresses, chilling injury and virus/viroid infection. By contrast, we identified over-represented functional categories and subcategories among the set of repressed genes belonging to each of the main GO domains (Table 2, threshold > 1.5 fold). We found genes involved in “response to external stimulus”, “defense response”, “catabolic processes” and “binding function or cofactor requirement”. Downregulation of these functions is presumably due to the virus counterattack against host defense-related pathways. Moreover, these host functions are frequently found in gene expression studies in response to virus infection [25].

By increasing the threshold to at least 2 fold changes we identified enriched functional categories only belonging to two of the main GO domains: Biological Process and Cellular Component (Table 2). Using this more stringent analysis we found groups of genes related with “response to stress”, to “external stimulus”, “amino acid metabolism” and “vacuole”. Importantly, the most overrepresented functional category was “response to water deprivation”. Among the genes assigned to this functional group genes related with “plant hormones” were found. Plant hormones play important roles in regulating developmental processes and signalling networks involved in plant responses to a wide range of biotic and abiotic stresses. For instance we identified a tonoplast resident H^+^-pump (At1g15690 H^+^-PPase AVP1) that contributes to vacuolar acidification, regulation of apoplastic pH and auxin transport [43]. Auxin acts as an important component of hormone signaling network involved in the regulation of defense responses against various biotrophic and necrotrophic pathogens. Additionally, this hormone regulates the expression of genes associated with the biosynthesis, catabolism and signaling pathways of other hormones [44]. Viral pathogens manipulate auxin signaling components to promote virulence and cause disease. One example is the interaction of *Tobacco mosaic virus* (TMV) replicase with Aux/IAA proteins. Development of symptoms promoted by this interaction has been described in Arabidopsis and tomato [45-47] and in addition disrupts the nuclear localization of several Arabidopsis Aux/IAA proteins. A second example is the protein phosphatase AtPP2CA (At3g11410) which acts as strong negative regulator of ABA signal transduction during seed germination and the regulation of stomatal closure [48]. Tobacco plants infected with TMV showed increased ABA levels and treatment with ABA enhanced TMV resistance in tobacco [49]. Some pathogens might have evolved the ability to produce ABA or ABA-mimicking substances to interfere with host defence. In any case, the role of ABA during plant-pathogen interactions depends on the individual plant-pathogen combination [50].

As members of the functional group “response to water deprivation” we also found proteins directly related with protection against oxidative stress like the Aldehyde dehydrogenase (At1g54100) and the Late embryogenesis abundant (LEA)-like protein (AtLEA5) (At4g02380). Aldehyde dehydrogenases (ALDHs) regulate the level of aldehydes by metabolizing excessive amounts of aldehyde molecules, which accumulate as a result of perturbed environmental conditions. Aldehydes are involved in different cellular metabolic processes but in excess they can have toxic effects on the cells. It is important then to maintain an appropriate balance of these molecules to avoid cellular damages. It has been described that over-expression of different ALDHs in *A. thaliana* confers tolerance to abiotic stress and protects plants against lipid peroxidation and oxidative stress [51]. (LEA)-like proteins protect other proteins from aggregation from desiccation. AtLEA5 is the unique which is induced specifically by reactive oxygen species (ROS) as well as by ABA but it is unlikely to act as an antioxidant enzyme. It has been suggested that LEA5 may cooperate with other factors to protect cellular components against ROS-induced damage or indeed to enhance the turnover of specific proteins during stress to enable rapid acclimation to the prevailing conditions [52].

In the few cases in which changes in the host transcriptome have been investigated upon double virus infections (e.g. the PVX-PVY interaction in *N. benthamiana*[24]) a severe oxidative stress was inferred to be induced in infected plant leaves, as increased transcript levels of genes encoding proteins important for lipid peroxidation for the generation of ROS were observed. We did not observe any significant over- or underrepresentation of genes involved in the antioxidative system in the doubly infected peach fruits. This difference between PLMVd-PNRSV-infected peach fruits and PVX-PVY infected N. benthamiana plants may be explained by the nature of the interaction between the two pathogens and the pathogens and the host. While co-infection of *N. benthamiana* with PVX-PVY can be considered as a true biological synergistic interaction leading to enhanced disease phenotypes compared to single infection with either pathogen, the doubly-infected PNRSV-PLMVd peach fruits did not exhibit an enhanced disease phenotype compared to single infection with either PNRSV or PLMVd. Interestingly, PNRSV was able to induce significant oxidative stress and an imbalance in the antioxidant systems in infected apricot-seeds [53] resulting in a decrease in seed germination. This observation indicates that host responses are not only specific for the host plant and the pathogen, but also tissue and/or organ-specific.

In the functional category “response to dehydration” which was strongly overrepresented upon double infection with PNRSV and PLMVd, we also found other important players with known functions in plant immunity. Among those was Cysteine protease RD21 (At1g47128), a Papain-like cysteine protease (PLCP). These proteins participate in immune responses and are targeted by pathogen- derived inhibitors. PLCPs are also required to trigger the hypersensitive response (HR) [54,55]. Consistently, PLCP RD19 is required in Arabidopsis for *RRS1-R-*mediated resistance against the bacterial pathogen *Ralstonia solanacearum* producing effector PopP2 [56]. A crucial role of PLCPs in disease immunity is also indicated by the observation that many pathogens produce effectors that manipulate these proteases [56-61].

## Conclusions

Our data reveal for the first time a clear synergistic effect between a viroid and plant virus at the transcriptome level. We demonstrate that mixed infections, which occur frequently in the field, can result in a more complex transcriptional response than that observed in single infections under the same experimental conditions. Thus, our analysis takes first steps to illuminate the mechanistic basis of synergistic mixed infections in peach trees and reveals candidate genes which can be tried as targets for crop protection. Future additional studies will further elucidate facilitative or antagonistic interactions between plant viruses/viroids in mixed infections.

## Methods

### Plant material

A total of 2,456 stone fruit trees were sampled during the whole survey period. Three trees were randomly sampled within each orchard. Twenty leaves per plant were collected from several points of the canopy and pooled leaves of each plant were tested for the presence of viruses and viroids previously reported from Chile, i.e. (PPV), (PDV), (PNRSV), (ACLSV), (ToRSV), (PLMVd) [62-65] and for viruses and viroids until now unrecorded in the country, i.e. *Apple mosaic virus* (ApMV), *American plum line pattern virus* (APLPV) and *Hop stunt viroid* (HSVd). Detection was carried out by nonradioactive molecular hybridization (MH) using the corresponding probes for the different viral/viroidal sequences [27,66]. Reverse transcription-polymerase chain reaction (RT-PCR) was used on selected positive and negative samples, and in all the samples which showed questionable and weak reactions using MH. Infected and healthy fruits were collected from the selected positive and negative samples and analysed using the same detection methods described above.

Due to the absence of a unique peach tree variety infected with all the suggested combinations of virus/viroid for this study, we used three different peach tree varieties. Kawea: for healthy and PLMVd infected samples, White Lady: for PNRSV infected samples and Rosario Red for doubly infected samples. Before proceeding with the microarray analysis, we checked whether the distinct varieties could influence in the transcriptome analysis. For this purpose, we used Real Time qRT-PCR and amplified several genes in different infection scenarios against the same healthy variety. No influence of the different varieties used on the transcriptome profile was observed.

### RNA extraction

Total RNA from peaches for subsequent functional analyses was extracted as described by Meisel et al. [67]. RNA quality and quantity was analyzed spectrophotometrically by evaluating the absorption ratios: A260/230 and A260/280 and confirmed with an EtBr stained 1.5% agarose gel containing 3% formaldehyde. Presence or absence of the different virus and viroids detected in above leaves was confirmed by MH. Positive tested samples infected either with PNRSV, PLMVd, or both pathogens, as well as healthy controls were selected for microarray profiling.

### Microarray hybridization, scanning and data analyses

For each biological replicate equal amounts of samples from four peach fruits belonging to four different trees were pooled and RNA was extracted. To obtain robust and statistically accurate data, four biological replicates and two technical replicates (dye swaps: Cy3-healthy/Cy5-infected or Cy3-infected/Cy5-healthy) were used for all the different samples.

Amplification of RNA samples for microarray hybridization was carried out using the method of Van Gelder et al., 1990 [68]. 1.25 μg of total RNA of each sample were amplified and aminoallyl-labelled using the MessageAmp® II aRNA kit (Ambion, http://www.ambion.com) and 5-(3-aminoallyl)-2´-deoxyuridine-5´-thiphosphate (aa-Dutp, Ambion), following manufacturer’s instructions. A rough amount of 20 μg of amplified RNA (aRNA) was obtained and 7.5 μg from each sample of aminoallyl-labelled aRNA were re-suspended in coupling buffer labelled with either Cy3 or Cy5 Mono NHS Ester (CyTM Dye Postlabelling Reactive Dye Pack, Amersham). Sample purification was carried out with MegaclearTM (Ambion) following manufacturer instructions. Incorporation of Cy3 and Cy5 was measured using 1 μl of the probe in a Nanodrop spectrophotometer (Nanodrop Technologies Inc.; http://www.nanodrop.com/).

Hybridization of samples and reference pool (RP) to the microarray slides was performed manually using Telechem Hybridization Chambers (Corning), according with the protocol described by Ogundiwin et al. [34] and manufacturer’s instructions. To detect differentially expressed genes in PNRSV, PLMVd or doubly infected samples in comparison to healthy samples, the data were analyzed with the SAM package (Significance Analysis of Microarray, [69]. Statistical significance was assessed using SAM analyses with a false discovery rate of 5% with no fold change cut-off. To study the functional category distribution of genes with expression changes we used the Munich Information Center for Protein Sequences (MIPS FunCatDB, http://mips.helmholtz-muenchen.de/proj/funcatDB/). Gene lists were further analyzed with FatiGO to find differential distributions of gene ontology (GO) terms between statistically differential expressed genes and the rest of genes present on the microarray (Fisher’s exact test in 2 x 2 contingency tables), with *P* values adjusted after correcting for multiple testing.

### Real-time qRT-PCR analysis

Nine genes found to be differentially expressed upon infection in the microarray experiments were selected for qRT-PCR analysis. qRT-PCR was performed with 100 ng total RNA using one step SYBR PrimeScript RT-PCR Kit II (Takara) following the manufacturer’s recommendations in an Applied Biosystems 7500 Real-Time PCR System. Each biological replicate was assayed in triplicate. Gene-specific oligonucleotide primers were designed using Primer Express® version 3.0 software (Applied Biosystems). Primer information is shown in Table 3. Expression levels for target genes were normalized to Elongation Factor 1-alpha (EF1 α) and fold expression changes compared to the healthy controls calculated using the DDthreshold cycle (Ct) method.

**Table 3:** **Real-time polymerase chain reaction primers, gene targets and the corresponding *Arabidopsis thaliana *homologs**

***Protein***	***Locus name (Peach)***	***Arabidopsis thaliana homolog***	***% Homology***	***Forward primer (5´-3´)***	***Reverse primer (5´-3´)***
Elongation factor 1-alpha (EF1-a)	ppa005718m.g	At5g60390	95.3	CCTTTGTCCCCATTTCTGGAT	CCTTTGTCCCCATTTCTGGAT
Full=F-box (F-BOX)	ppa005877m.g	At5g15710	84.6	CCTTTGTCCCCATTTCTGGAT	AGGATGAATTGCTTTGCCAAA
Clathrin adapter complex (CAC)	ppa005912m.g	At5g46630	90.9	CAAAATTCCTGTGCCAAAACAA	GCTCGACCCGAAGTCACTTG
Expansin (EXP8)	ppa010260m.g	At2g40610	78.7	TGGTGGGTGGTGCAATCC	AGAAAGCAGGCTCAGCCAAA
Phytoene synthas	ppa005962m.g	At5g17230	81.5	TGGGCCTAACGCCTCACA	TCTTCTAACCTCGACTCCCACCTA
Auxine response protein (IAA9)	ppa007194m.g	At5g65670	71.3	TGATTCATGCAAGAGGTTGAAGA	GCCCTAGGAGCTAAGCCAATG
Glutamate descarboxilase	ppa004796m.g	At5g17330	89.0	TGAAGGCTGCCGATGGA	TACTCTCAAGTGCCCTCGTCTCT
Cysteine proteinase	ppa004796m.g	At1g47128	80.3	CAACCATGGCGATTCTTTTTC	ATTGACATGTCCACGGCTGAT
Invertase/pectin methylesterase inhibitor family	ppa011831m.g	At5g62350	64.4	CCTGCCTTATGTGTCCACTCACT	AAACGTTTAGGGCTTGTTTGGAT
Glutamate deshydrogenase	ppa006458m.g	At5g07440	95.4	TCGATTCAGGGTTTGACATTTGT	GCTTCGCAGCCCATGTTC
Universal Stress Protein	ppa012560m.g	At3g53990	85.6	TCGGAATCGCCATGGATTT	CTCGATCGCCCATTTCAGA
CBL-interacting protein Kinase 6	ppa006023m.g	At4g30960	85.7	GCTTCACGGCCGTTACGAT	GTGGTACACCTTCGCGAATGT

### Dot-blot hybridization

Total nucleic acids were first denatured with formaldehyde, serially diluted [36] and then applied to nylon membranes (positive charged, ROCHE), air dried and covalently UV cross-linked to the membrane (700 × 100 μJ/cm^2^). Prehybridizations and hybridizations with specific probes for the specific viruses and viroids were conducted as described previously [28,70]. Chemiluminiscent detection with CSPD reagent as the substrate was performed as recommended by the manufacturer (ROCHE).

## Competing interests

The authors declare that they have no competing interests.

## Authors’ contributions

Conceived and designed the experiments: MCH and VP. Performed the transcriptomic experiments: MCH. Performed the viral diagnosis: AG, NF, AZ, MCH and MR. Analyzed the data: MCH, AN and VP. Wrote the paper: MCH, AN and VP. All authors discussed the results and commented on the manuscript. All authors read and approved the final manuscript.

## Supplementary Material

Additional file 1: Figure S1 Comparison between amounts of PLMVd and PNRSV in infected samples used for profiling. Average ± standard deviation (SD) of pixel intensity corresponding to PLMVd or PNRSV titer at a concentration of 0.3ng RNA (see Figure 2). Virus/viroid titer in samples infected with PLMVd (left graph) or PNRSV (right graph) was slightly lower compared to virus/viroid titer in samples infected with both pathogens simultaneously. Click here for file

Additional file 2: Table S1 Genes with significantly altered expression upon the three different infections. Between brackets the number of genes with orthologs in Arabidopsis is shown. Click here for file

Additional file 3: Table S2 Genes with significant expression changes and orthologs in Arabidopsis. Click here for file

Additional file 4: Table S3 Common genes differentially expressed upon each infection scenario. According to the Venn diagrams (Figure 4) the table shows the description of the genes with altered expression which are common between the different combinations of infection. Between brackets the number of genes for each scenario is shown. Click here for file

Additional file 5: Table S4 Genes related with ripening process and with significantly altered expression upon the three different infections. Expansin putative (EXP1), At1g69530; Glutamate dehydrogenase 2 (GDH2), At5g07440; Expansin, putative (EXP8), At2g40610; Auxin-responsive protein / indoleacetic acid-induced protein 9 (IAA9), At5g65670; bZIP transcription factor family protein, similar to common plant regulatory factor 7, At4g34590; Glutamate dehydrogenase 2 (GDH2), At5g07440; Auxin-responsive protein / indoleacetic acid-induced protein 17 (IAA17), At1g04250; Invertase/pectin methylesterase inhibitor family protein, At5g62350; Agamous-like MADS box protein AGL8, At5g60910; Zinc finger (C3HC4-type RING finger) family protein, At5g25560. Click here for file
