# Supplementary material for: A remarkable synergistic effect at the transcriptomic level in peach fruits doubly infected by prunus necrotic ringspot virus and peach latent mosaic viroid
Source: Virol J. 2013 May 28;10:164. doi: 10.1186/1743-422X-10-164 (PMC3672095; doi:10.1186/1743-422X-10-164)
Supplement: Additional file 1: Figure S1 — Comparison between amounts of PLMVd and PNRSV in infected samples used for profiling. Average ± standard deviation (SD) of pixel intensity corresponding to PLMVd or PNRSV titer at a concentration of 0.3ng RNA (see Figure 2). Virus/viroid titer in samples infected with PLMVd (left graph) or PNRSV (right graph) was slightly lower compared to virus/viroid titer in samples infected with both pathogens simultaneously. [file 1743-422X-10-164-S1.ppt]

## Slide 1
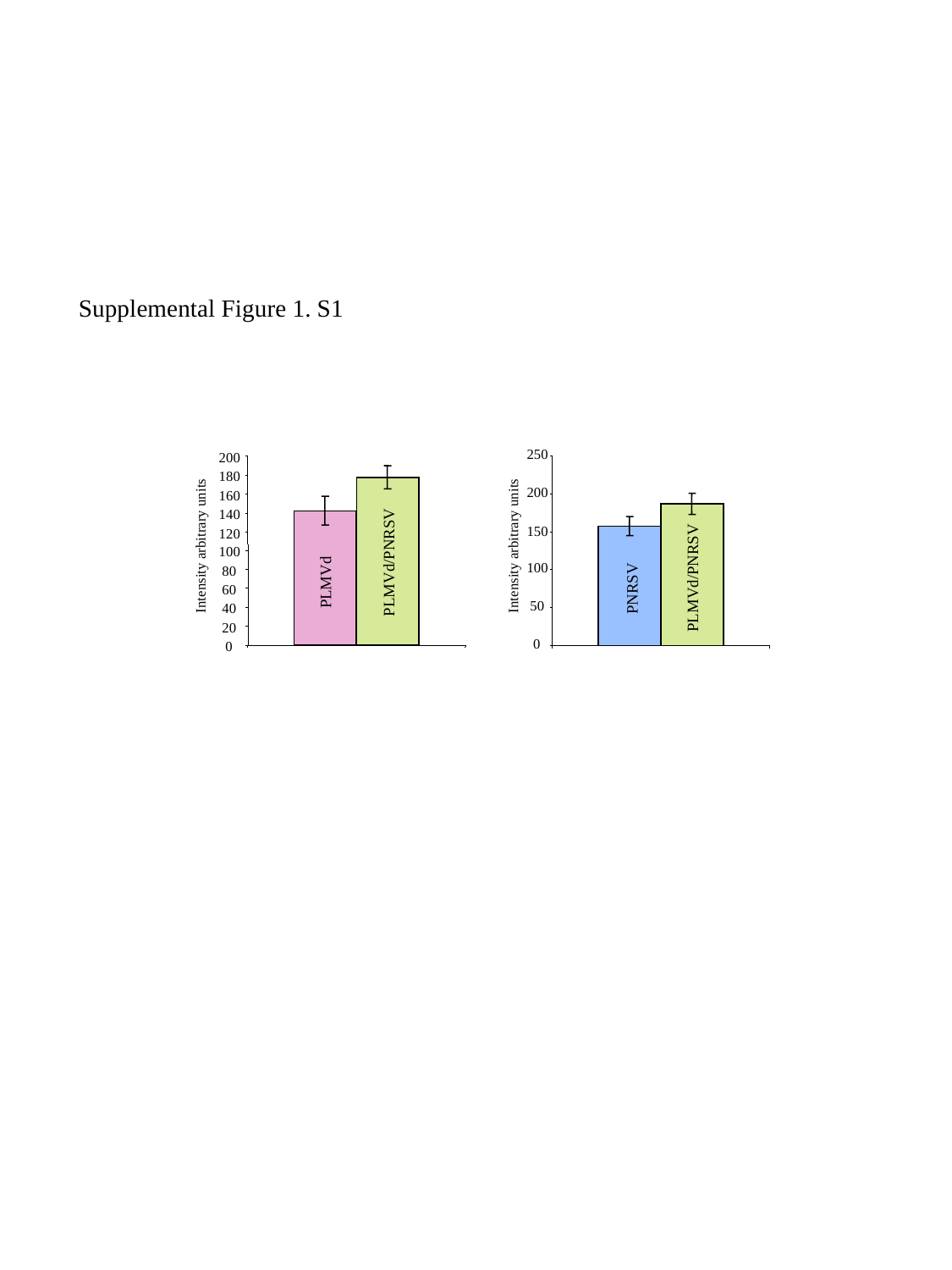

Supplemental Figure 1. S1
250
200
180
200
160
140
150
120
Intensity arbitrary units
Intensity arbitrary units
100
PLMVd/PNRSV
100
80
PLMVd/PNRSV
PLMVd
PNRSV
60
50
40
20
0
0
