# Supplementary material for: A remarkable synergistic effect at the transcriptomic level in peach fruits doubly infected by prunus necrotic ringspot virus and peach latent mosaic viroid
Source: Virol J. 2013 May 28;10:164. doi: 10.1186/1743-422X-10-164 (PMC3672095; doi:10.1186/1743-422X-10-164)
Supplement: Additional file 2: Table S1 — Genes with significantly altered expression upon the three different infections. Between brackets the number of genes with orthologs in Arabidopsis is shown. [file 1743-422X-10-164-S2.pptx]

## Slide 1
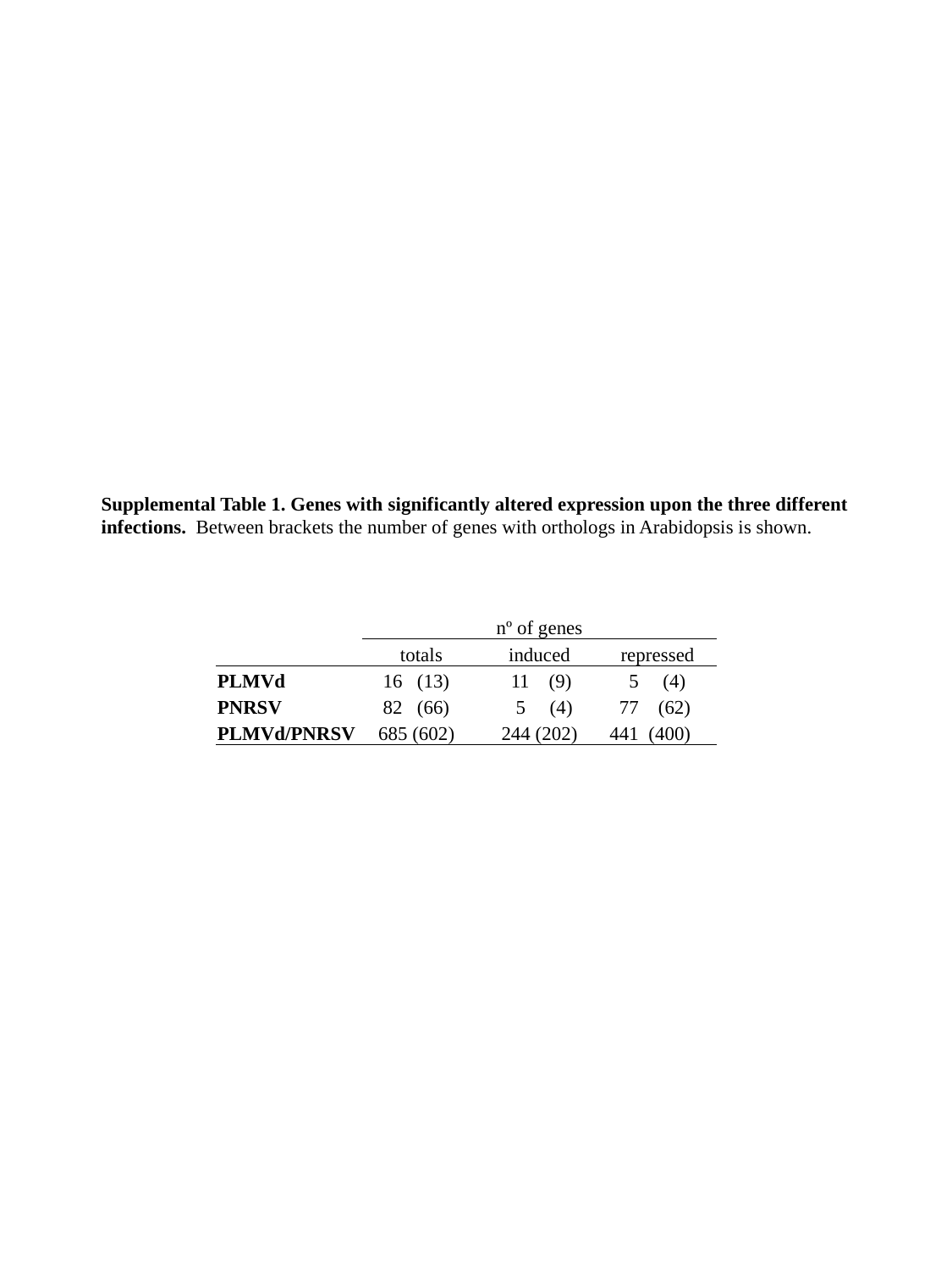

Supplemental Table 1. Genes with significantly altered expression upon the three different
infections. Between brackets the number of genes with orthologs in Arabidopsis is shown.
| | | nº of genes | |
| --- | --- | --- | --- |
| | totals | induced | repressed |
| PLMVd | 16 (13) | 11 (9) | 5 (4) |
| PNRSV | 82 (66) | 5 (4) | 77 (62) |
| PLMVd/PNRSV | 685 (602) | 244 (202) | 441 (400) |
