# Supplementary material for: A remarkable synergistic effect at the transcriptomic level in peach fruits doubly infected by prunus necrotic ringspot virus and peach latent mosaic viroid
Source: Virol J. 2013 May 28;10:164. doi: 10.1186/1743-422X-10-164 (PMC3672095; doi:10.1186/1743-422X-10-164)
Supplement: Additional file 5: Table S4 — Genes related with ripening process and with significantly altered expression upon the three different infections. Expansin putative (EXP1), At1g69530; Glutamate dehydrogenase 2 (GDH2), At5g07440; Expansin, putative (EXP8), At2g40610; Auxin-responsive protein / indoleacetic acid-induced protein 9 (IAA9), At5g65670; bZIP transcription factor family protein, similar to common plant regulatory factor 7, At4g34590; Glutamate dehydrogenase 2 (GDH2), At5g07440; Auxin-responsive protein / indoleacetic acid-induced protein 17 (IAA17), At1g04250; Invertase/pectin methylesterase inhibitor family protein, At5g62350; Agamous-like MADS box protein AGL8, At5g60910; Zinc finger (C3HC4-type RING finger) family protein, At5g25560. [file 1743-422X-10-164-S5.pptx]

## Slide 1
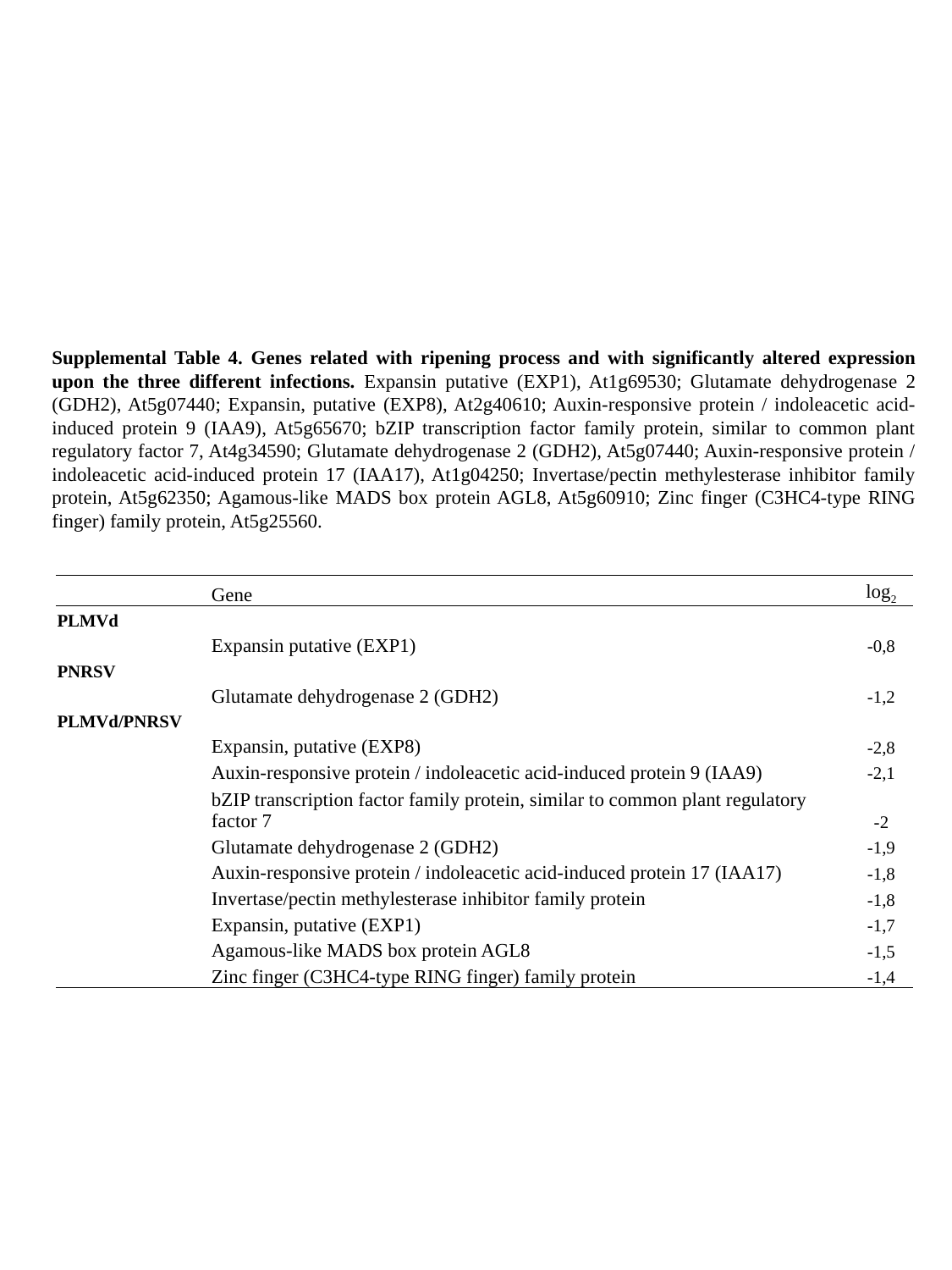

Supplemental Table 4. Genes related with ripening process and with significantly altered expression upon the three different infections. Expansin putative (EXP1), At1g69530; Glutamate dehydrogenase 2 (GDH2), At5g07440; Expansin, putative (EXP8), At2g40610; Auxin-responsive protein / indoleacetic acid-induced protein 9 (IAA9), At5g65670; bZIP transcription factor family protein, similar to common plant regulatory factor 7, At4g34590; Glutamate dehydrogenase 2 (GDH2), At5g07440; Auxin-responsive protein / indoleacetic acid-induced protein 17 (IAA17), At1g04250; Invertase/pectin methylesterase inhibitor family protein, At5g62350; Agamous-like MADS box protein AGL8, At5g60910; Zinc finger (C3HC4-type RING finger) family protein, At5g25560.
| | Gene | log2 |
| --- | --- | --- |
| PLMVd | | |
| | Expansin putative (EXP1) | -0,8 |
| PNRSV | | |
| | Glutamate dehydrogenase 2 (GDH2) | -1,2 |
| PLMVd/PNRSV | | |
| | Expansin, putative (EXP8) | -2,8 |
| | Auxin-responsive protein / indoleacetic acid-induced protein 9 (IAA9) | -2,1 |
| | bZIP transcription factor family protein, similar to common plant regulatory factor 7 | -2 |
| | Glutamate dehydrogenase 2 (GDH2) | -1,9 |
| | Auxin-responsive protein / indoleacetic acid-induced protein 17 (IAA17) | -1,8 |
| | Invertase/pectin methylesterase inhibitor family protein | -1,8 |
| | Expansin, putative (EXP1) | -1,7 |
| | Agamous-like MADS box protein AGL8 | -1,5 |
| | Zinc finger (C3HC4-type RING finger) family protein | -1,4 |
